# *Helq* acts in parallel to *Fancc* to suppress replication-associated genome instability

**DOI:** 10.1093/nar/gkt676

**Published:** 2013-08-21

**Authors:** Spencer W. Luebben, Tsuyoshi Kawabata, Monica K. Akre, Wai Long Lee, Charles S. Johnson, M. Gerard O’Sullivan, Naoko Shima

**Affiliations:** ^1^Department of Genetics, Cell Biology and Development, University of Minnesota, Minneapolis, MN 55455, USA, ^2^Molecular, Cellular, Developmental Biology and Genetics Graduate Program, University of Minnesota, Minneapolis, MN 55455, USA, ^3^Masonic Cancer Center, Minneapolis, MN 55455, USA and ^4^College of Veterinary Medicine, University of Minnesota, St. Paul, MN 55108, USA

## Abstract

HELQ is a superfamily 2 DNA helicase found in archaea and metazoans. It has been implicated in processing stalled replication forks and in repairing DNA double-strand breaks and inter-strand crosslinks. Though previous studies have suggested the possibility that HELQ is involved in the Fanconi anemia (FA) pathway, a dominant mechanism for inter-strand crosslink repair in vertebrates, this connection remains elusive. Here, we investigated this question in mice using the *Helq^gt^* and *Fancc*^−^ strains*.* Compared with *Fancc*^−^*^/^*^−^ mice lacking FANCC, a component of the FA core complex, *Helq^gt/gt^* mice exhibited a mild of form of FA-like phenotypes including hypogonadism and cellular sensitivity to the crosslinker mitomycin C. However, unlike *Fancc*^−^*^/^*^−^ primary fibroblasts, *Helq^gt/gt^* cells had intact FANCD2 mono-ubiquitination and focus formation. Notably, for all traits examined, *Helq* was non-epistatic with *Fancc*, as *Helq^gt^^/gt^;Fancc*^−^*^/^*^−^ double mutants displayed significantly worsened phenotypes than either single mutant. Importantly, this was most noticeable for the suppression of spontaneous chromosome instability such as micronuclei and 53BP1 nuclear bodies, known consequences of persistently stalled replication forks. These findings suggest that mammalian HELQ contributes to genome stability in unchallenged conditions through a mechanism distinct from the function of FANCC.

## INTRODUCTION

Maintaining genome stability is critical for cells, given that the genome is under constant attack by numerous exogenous and endogenous agents (1). It is inevitable that cells will have to proceed with genome duplication using damaged template DNA, which can perturb the normal progression of replication forks. To cope with this problem, organisms have developed multiple mechanisms to allow the rescue of stalled replication forks (2). Such mechanisms include firing of dormant origins, translesion synthesis with error-prone DNA polymerases and homology-directed fork recovery (3–5). Evidence suggests that the latter two mechanisms are in part coordinated by the concerted work of proteins that are mutated in Fanconi anemia (FA) (6), a rare polygenic human genetic disorder (7). FA patients exhibit genome instability, congenital abnormalities, bone marrow failure, hypogonadism and a heightened predisposition to cancer (7,8). FA is uniquely characterized by its cellular hypersensitivity to agents that induce DNA inter-strand crosslinks (ICLs) (7,9), although it is unclear how ICL repair is functionally linked with stalled fork recovery in unchallenged conditions.

The DNA helicase HELQ was first discovered in the human and mouse genomes through its homology to MUS308 (10), a DNA repair enzyme required for ICL resistance in *Drosophila melanogaster* (11,12). Although it is unlikely that its vertebrate ortholog POLQ plays a major role in ICL repair (13–15), together they make up a unique family of DNA polymerases that possess a helicase domain in the N-terminus in addition to a C-terminal polymerase domain (16–18). Unlike its paralog POLQ, HELQ lacks a polymerase domain, and several lines of evidence indicate that HELQ performs a distinct function from POLQ. *HELQ* is an ortholog of the Drosophila *mus301* gene (19), which is allelic to the female-sterile mutation *spindle-C* (*spn-C*) (20). Mutations in *spn-C* result in the failed repair of meiotic double-strand breaks (DSB) and activation of the meiotic checkpoint (20), which was not observed in *mus308* mutants. In line with this observation, it was also reported that the *Caenorhabditis elegans* ortholog *helq-1* plays a role in meiotic DSB repair by promoting postsynaptic RAD-51 filament disassembly (21). These findings suggest that HELQ has a role in meiotic DSB repair through homologous recombination (HR) in these species. In humans, *HELQ* is expressed in the testes, ovaries, heart and skeletal muscle (22). However, its function is largely unknown.

Biochemically, human HELQ exhibits ATP-dependent 3′–5′ DNA helicase activity *in vitro* (10,23). A recent study demonstrated that human HELQ preferentially unwinds the parental strands of forked structures with a nascent lagging strand, and that this activity is stimulated by replication protein A (RPA) (23). These findings suggest that HELQ is likely to participate in the recovery of stalled or collapsed replication forks. Several studies have suggested that this role of HELQ is closely linked with the FA pathway. A genetic study in *C. elegans* demonstrated that *helq-1* is required for ICL repair and is epistatic to *fcd-2* (24), an ortholog of *FANCD2* whose product is mono-ubiquitinated by the FA core complex as a key step in this pathway (25). However, *C. elegans* contains only a few FA proteins and lacks multiple members comprising the FA core complex (26). HELQ may belong to a primitive FA pathway in *C. elegans*, but its evolution seems to have taken a complex path. Paradoxically, disruption of *Helq* in chicken DT40 cells, which contain all of the FA proteins, did not confer hypersensitivity to ICL inducing agents (14). In human cells, HELQ depletion confers hypersensitivity to the crosslinker mitomycin C (MMC) and HR deficiency, the latter reported to be epistatic to FANCD2 (27). Consistent with this observation, exogenously expressed GFP-tagged HELQ co-localizes with RAD51 foci as well as FANCD2 foci after treatment with the topoisomerase I inhibitor camptothecin (CPT) (23). There is little information about the link between HELQ and the FA pathway in mammals, particularly in the absence of exogenous DNA damage.

To decipher the enigmatic connection between HELQ and the FA pathway, we have generated *Helq* deficient mice using a gene-trap allele named *Helq^gt^* for phenotypic comparisons to mice deficient for *Fancc*, encoding FANCC, a component of the FA core complex (28) in the same genetic background. For all traits examined including hypogonadism and MMC sensitivity, we found that loss of *Helq* results in phenotypes considerably milder than *Fancc* deficiency. Moreover, our data show that combined loss of *Helq* and *Fancc* leads to further severe phenotypes than single mutants, presenting no evidence for epistasis. Importantly, the strongest inter-dependence for *Helq* and *Fancc* was observed for the suppression of spontaneous genome instability derived from replication fork failures rather than MMC resistance. These findings collectively suggest that HELQ contributes to genome stability in unperturbed conditions in a manner that is distinct from the function of FANCC.

## MATERIALS AND METHODS

### Mouse strains and mouse embryonic fibroblasts

All experiments were performed using mice from a C57BL/6J background and were approved by the Institutional Animal Care and Use Committee. Mouse embryonic fibroblasts (MEFs) were generated from 12.5–14.5 dpc embryos and cultured using standard procedures as described previously (29). All mice were genotyped by PCR. The primers used are available upon request.

### Quantitative reverse-transcription-PCR

RNA was isolated from either cultured MEFs or testes tissue using the PureLink RNA Mini Kit (Ambion, Life Technologies) and the RNeasy Kit (QIAGEN). cDNA was then synthesized using the Superscript VILO cDNA Synthesis Kit (Invitrogen, Life Technologies). q-PCR analysis was performed on the LightCycler 480 (Roche) using primer pairs specific for exons 1–2, exons 11–12 and the chimeric mutant transcript spanning between exon 11 and the inserted vector. Expression was normalized to glyceraldehyde 3-phosphate dehydrogenase (GAPDH).

### Western blotting and immunofluorescence microscopy

Western blotting and immunofluorescence staining were carried out using standard procedures as described previously (29).

### Antibodies

For immunofluorescence and western blotting procedures, we used anti-phospho-histone H3, anti-RPA32, anti-γH2AX, anti-CENP-A, anti-phospho-CHK1 (Cell Signaling; #9706, #2208, #2577, #2048, #2341, respectively), anti-FANCD2 for foci staining, anti-FANCI, anti-53BP1, anti-MCM4 (Abcam; ab2187 or ab108928, ab74332, ab36823, ab4459, respectively), anti-FANCA (Bethyl Laboratories; #A301-980A), anti-CHK1 (Santa Cruz; sc-8408), anti-FANCD2 for western blots (Epitomics; #2986-1) and anti-HELQ (MyBioSource; #MBS120320). For the DNA fiber assay, anti-digoxigenin antibody conjugated with rhodamine from Roche (11207750910) and the streptavidin-AlexaFluor488 from Invitrogen (S-32354) were used.

### siRNA transfection in MEFs, HEK 293T and PD331 cells

One million cells were seeded per well in a six-well dish followed by transfection with either 50 (MEFs) or 25 nM (HEK 293T, PD331) of non-targeted control small interfering RNA (siRNA) (#D-001206-13-20, siGENOME Smart pool), HELQ siRNA (#M-015379-01-0005, siGENOME Smart pool) or FANCA siRNA (#M-019283-02-0005, siGENOME Smart pool) from Dharmacon. This was performed using OPTI-MEM and Lipofectamine RNAiMAX (Life technologies) transfection reagents. Twenty-four hours later, a second round of transfection was performed using the same concentration of siRNA, followed by 24 h culture. Cells were then re-plated according to the analysis performed. The PD331 cell lines were obtained from the Oregon Health & Science University Fanconi Anemia Cell Repository (Portland, Oregon).

### Metaphase analysis

MEFs were treated with 600 nM MMC for 2 h and allowed 22 h to recover before harvest. For experiments using the HEK 293T cell line, cells were treated with 300 nM MMC for 24 h before harvest. In all experiments, cells were treated with colcemid for 1–2 h before harvest. Following hypotonic treatment, cells were fixed with fixative (3:1 methanol: acetic acid in volume) and dropped on slides in a humidified environment to optimize spreading. Slides were mounted in 1× 4′,6-diamidino-2-phenylindole, dihydrochloride (DAPI) anti-fade solution the following day and blinded for analysis. Only metaphases with 38–41 chromosomes (MEFs) or 64–72 chromosomes (HEK 293T cells) were included in the analysis. Chromosome aberrations including radials, gaps/breaks, fragments and ring chromosomes were scored for all experiments. HEK 293T cells were cultured using the same procedures used for MEFs.

### DNA fiber assay

All techniques and methods of analysis used were performed as described previously (29,30). Briefly, replication forks were sequentially labeled with deoxyuridine triphosphates (dUTPs) conjugated with digoxigenin (digoxigenin-dUTPs) for 20 min and with biotin-dUTPs for 30 min. Labeled cells were dropped onto slides, fixed and dipped into lysis buffer for the release and extension of DNA fibers. Incorporated dUTPs were visualized by anti-digoxigenin rhodamine conjugate (Roche, Branford, CT) and streptavidin, -Alexa Fluor 488 (Invitrogen, Carlsbad, CA).

### Colony formation assay

Five hundred cells were plated in 6 cm dishes along with the corresponding doses of MMC. For HEK 293T cells, the bottoms of the dishes were coated with poly-L-lysine beforehand to aid in cell adhesion. Colonies were stained using crystal violet after a period of 1 week (HEK 293T) or 2 weeks (PD331 and PD331+FANCC) and counted.

### MTT assay

The Vybrant MTT (3-(4,5-dimethylthiazol-2-yl)-2,5-diphenyltetrazolium bromide) Cell Proliferation Assay Kit (Life Technologies) was used. Briefly, 5 × 10^4^ (experiments with MEFs) or 1 × 10^4^ (experiments with PD331 cell lines) cells of each genotype were plated per well in a 96-well plate. The next day, cells were either treated with the corresponding drug or left untreated for 5 days before the assay was performed according to the manufacturer’s instructions. A_570_ was used to measure relative cell proliferation, whereas A_670_ was used as a reference for background absorbance.

### Cytokinesis-block micronucleus assay and G1 phase cell analyses

The cytokinesis-block micronucleus (MN) assay was performed as described previously (29,31), except that cells were treated with cytochalasin B (0.72 µg/ml) for 4–5 h. The same procedure was used for all analyses of G1 cells.

### Measuring HR events using the fluorescent yellow direct repeat transgenic locus system

Wild-type (WT) and *Helq^gt/gt^* MEFs carrying the fluorescent yellow direct repeat (*FYDR*) transgenic locus (32) in the hemizygous state were generated as described earlier in the text. Cells at passage 2 were plated, grown for 3 days and then re-plated into three separate dishes. The corresponding drug treatments (untreated, MMC, CPT) were then administered the following day and washed out after 24 h. After a 48 h recovery period, cells were analyzed by flow cytometry using the FL1-H and FL2-H channels of the FACSCalibur (BD Biosciences).

## RESULTS

### *Helq* deficient mice carry a gene-trap allele that generates HELQΔ-β-Geo

**Figure 1.**
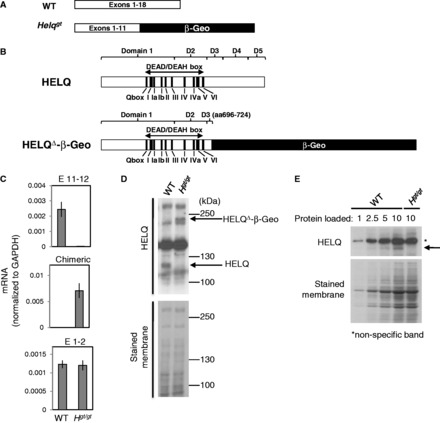
Characterization of the *Helq^gt^* allele. Diagrams (drawn to scale) depicting the transcripts (**A**) and resulting peptides (**B**) for the WT *Helq* and *Helq^gt^* alleles. The HELQ protein is split into five domains, the first two of which contain the well conserved motifs of the DEAD/DEAH helicase box. The *Helq^gt^* allele has most of domain 3 and all of domains 4 and 5 replaced by β-Geo. (**C**) qRT-PCR analysis using total RNA extracted from WT or *Helq^gt/gt^* (*H^gt/gt^*) MEFs is shown. Data for the WT transcript containing exons 11 and 12 (top), the chimeric transcript containing exon 11 and the gene-trap vector sequence (middle) and transcript containing exons 1 and 2 upstream of the insertion site (bottom) are shown. *Helq^gt/gt^* MEFs have less than 1/100th of the levels of the WT *Helq* mRNA compared with WT cells (see top). Experiments were duplicated using RNA samples from different MEF lines to confirm reproducibility. A representative qRT-PCR data set is shown. (**D**) Western blotting shows no detectable levels of WT HELQ protein in *Helq^gt/gt^* MEFs. A band corresponding to the HELQ^Δ^-β-Geo fusion peptide appears at the predicted molecular weight of ∼230 kDa only in the lysate from *Helq^gt/gt^* cells. (**E**) Loading differing ratios of protein reveals that the amount of WT HELQ protein (indicated by arrow) is extremely low in *Helq^gt/gt^* MEFs. Asterisk indicates non-specific bands. A stained membrane was used as a loading control for (D) and (E).

To generate a mouse *Helq* mutant, we searched the BayGenomics database for mutant mouse embryonic stem (ES) cell clones (33,34). Gene-trap vectors are designed to have a splice acceptor site upstream of a reporter gene, typically β-Geo, a fusion gene of the β-galactosidase and neomycin resistance genes. We found that the ES cell clone RRF112 has an insertion of the gene-trap vector pGT0Lxf in the intron between exons 11 and 12 of the *Helq* locus (Supplementary Figure S1A). This gene-trap allele was named *Helq^gt^*. The *Helq^gt^* allele is expected to create a chimeric transcript containing exons 1–11 of *Helq* and β-Geo (Figure 1A), producing a truncated HELQ protein that is fused with β-Geo at its C-terminal end (Figure 1B)*.* This mutant protein still retains its helicase domain but lacks the three C-terminal domains with highly conserved motifs thorough archaea to metazoans (35,36). It has been demonstrated that these domains are required for normal helicase activity in other species (36–38). To generate mice that carry the *Helq^gt^* allele, we microinjected RRF112 ES cells into blastocysts from an inbred strain of C57BL/6J (B6) females using a standard method. High-percentage chimera males were mated with B6 inbred females to produce carriers of the *Helq^gt^* allele, which were identified by genomic PCR using primer pairs that amplify the boundary sequences of the insertion site (Supplementary Figure S1B). *Helq^gt^* heterozygous (*Helq^gt/+^*) carriers appear normal in every aspect and are indistinguishable from WT mice (data not shown). A congenic line of *Helq^gt^* has been established by backcrossing *Helq^gt/+^* mice to inbred B6 mice at least 10 generations. This B6 congenic *Helq^gt^* line was used for the following studies unless otherwise indicated.

### WT *Helq* mRNA and protein are virtually undetectable in *Helq^gt^* homozygous cells

*Helq^gt/+^* mice were timed-mated to generate MEFs. We extracted total RNA from WT and *Helq^gt^* homozygous (*Helq^gt/gt^*) MEFs for reverse-transcription (RT)-PCR and verified the presence of the chimeric message in *Helq^gt/gt^* MEFs and the WT mRNA in WT MEFs (Figure 1A) by sequencing the RT-PCR products (Supplementary Figure S1C). Furthermore, quantitative (q-)RT-PCR on RNA from WT and *Helq^gt/gt^* cells revealed that the WT transcript containing exons 11–12 was nearly absent in *Helq^gt/gt^* cells (<1% of WT) that predominantly express the chimeric transcript containing exon 11 and β-Geo sequences (Figure 1C). As we found no difference between WT and *Helq^gt/gt^* cells for the levels of transcript containing exons 1–2 far upstream of the insertion site, the presence of the gene-trap vector likely has no effect on *Helq* expression. Next, we performed western blots on whole-cell extracts from WT and *Helq^gt/gt^* MEFs (Figure 1D). Consistent with the q-RT-PCR results, WT HELQ (∼120 kD) was undetectable in *Helq^gt/gt^* cells. Instead, they predominantly express the mutant HELQ protein (HELQΔ-β-Geo). A semi-quantitative analysis indicated that *Helq^gt/gt^* cells express WT HELQ protein <10% of the levels seen in WT cells, if any at all (Figure 1E). These data suggest that the splice acceptor site at the *Helq^gt^* allele is efficient, leading to little expression of normal full-length HELQ in *Helq^gt/gt^* cells.

### *Helq* deficiency causes a mild form of hypogonadism, which is not epistatic to *Fancc*

**Figure 2.**
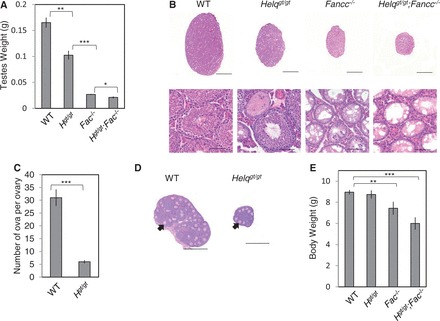
*Helq^gt/gt^* mice exhibit a hypogonadism phenotype reminiscent of mouse models of FA. (**A**) *Helq^gt/gt^*, *Fancc*^−^*^/^*^−^ and *Helq^gt/gt^;Fancc*^−^*^/^*^−^ males show significantly reduced testes weights at 6 weeks of age. At least five males were observed per genotype. (**B**) Histological analysis by hematoxylin and eosin staining shows a mosaic pattern of normal and empty seminiferous tubules in *Helq^gt/gt^*, *Fancc*^−^*^/^*^−^ and *Helq^gt/gt^;Fancc*^−^*^/^*^−^ mice, with the respective phenotypes becoming increasingly worse. Scale bars are 1500 µm for the whole testis sections (top) and 75 µm for the enlarged images (bottom). (**C**) *Helq^gt/gt^* females exhibit smaller ovaries with a reduced number of follicles at 3 weeks of age. Six ovaries from three WT females and 10 ovaries from five *Helq^gt/gt^* females were observed. (**D**) Hematoxylin and eosin staining of ovaries from WT and *Helq^gt/gt^* females. Example ova-containing follicles are indicated by arrows. Scale bars are 500 µm. (**E**) *Helq^gt/gt^* mice display normal body weights at weaning age, unlike *Fancc*^−^*^/^*^−^ and *Helq^gt/gt^;Fancc*^−^*^/^*^−^ mice, which are significantly smaller. In (A), (C) and (E), error bars represent the standard error of the means (SEMs) and significance was determined by *t*-test. Statistical significance at *P* < 0.05, *P* < 0.01, and *P* < 0.001 are indicated as *, ** and ***, respectively. WT, *H^gt/gt^*, *Fac*^−^*^/^*^−^ and *H^gt/gt^;Fac*^−^*^/^*^−^ refer to WT, *Helq^gt/gt^*, *Fancc*^−^*^/^*^−^ and *Helq^gt/gt^;Fancc*^−^*^/^*^−^, respectively.

As previous studies suggested an intriguing connection of HELQ to the FA pathway (23,24,27), we generated *Helq^gt/gt^* mice along with WT control mice to examine hypogonadism, one of the most consistent phenotypes seen in the FA mouse models (39). We found that *Helq^gt/gt^* males have significantly smaller testes (*P* < 0.005, *t*-test), ∼62% of WT males by weight at 6 weeks of age (0.165 ± 0.01 g and 0.102 ± 0.01 g for the average WT and *Helq^gt/gt^* testes weights, respectively, Figure 2A). Histological analysis revealed that ∼10–20% seminiferous tubules in *Helq^gt/gt^* males are atrophied and devoid of spermatocytes and spermatogonia at this stage (Figure 2B). This mosaic pattern of normal and empty seminiferous tubules is similar to what has been seen in a number of FA mouse models (40–48). For comparison, we also generated mice homozygous for a *Fancc* allele (*Fancc*^−^) (28) in the same background. As reported earlier (28,49,50), *Fancc*^−^*^/^*^−^ males had extremely small testes weighing an average of only 0.027 ± 0.001 g (only 16% of WT by weight, *P* < 0.0001, *t*-test) with >90% of seminiferous tubules exhibiting atrophy or hypotrophy (Figure 2A and B). When compared with *Helq^gt/gt^* males, *Fancc*^−^*^/^*^−^ testes were only 26% of *Helq^gt/gt^* testes by weight (*P* < 0.0001, *t*-test). These data suggest that the hypogonadism observed in *Helq^gt/gt^* testes is not as severe as in *Fancc*^−^*^/^*^−^ testes. To test for an epistatic relationship between *Helq* and *Fancc* for this trait, we generated mice doubly homozygous for *Helq^gt^* and *Fancc*^−^. Testes from *Helq^gt/gt^*;*Fancc*^−^*^/^*^−^ males were even smaller (average of 0.021 ± 0.002 g) than those from *Fancc*^−^*^/^*^−^ males (*P* < 0.05, *t*-test), having all seminiferous tubules completely devoid of spermatogonia and spermatocytes. Although *Fancc*^−^*^/^*^−^ and *Helq^gt/gt^*;*Fancc*^−^*^/^*^−^ mice are significantly smaller in size than WT and *Helq^gt/gt^* mice, these observations still hold true after taking this into consideration (Supplementary Figures S2A and B). Collectively, these findings indicate that mutations in *Helq* and *Fancc* are not epistatic to each other in causing hypogonadism.

### Female-specific sub-fertility in *Helq^gt/gt^* mice is consistent with germ cell hypoplasia during embryogenesis

It has been reported that hypogonadism in FA mouse models is attributed to severely compromised proliferation of primordial germ cells (40,41,45,51). This leads to sterility in a significant fraction of *Fancc*^−^*^/^*^−^ mice (28,49). However, as hypogonadism in *Helq^gt/gt^* males is modest, they are fertile, producing litters at sizes comparable with *Helq^gt/+^* males (Supplementary Table S1). Younger *Helq^gt/gt^* males have a greatly increased fraction (>50%) of seminiferous tubules exhibiting atrophy or hypotrophy (Supplementary Figure S2C). However, as they get older, the number of such tubules decreases. This is most likely because surviving germ cells in *Helq^gt/gt^* males can repopulate tubules as spermatogonial stem cells and support fertility as previously seen in FA mouse models (42). Therefore, much like the FA genes, *Helq* is required for normal proliferation of germ cells during embryogenesis but has no effect on spermatogonial stem cells at later stages. Different from males, *Helq^gt/gt^* females were more severely affected with hypogonadism (see Figure 2C and D). The number of ova per ovary was reduced to 6 ± 0.61 in *Helq^gt/gt^* females compared with 31 ± 3.2 in WT females (*P* < 0.0001, *t*-test). When initially tested in a 129/B6 mixed background, four of seven *Helq^gt/gt^* females were sterile. Fertile *Helq^gt/gt^* females tended to have small litters, with an average litter size of only 3.5 (n = 6). This female-specific sub-fertility is consistent with germ cell hypoplasia during embryogenesis, as it is believed that the total oocyte pool is determined at this stage. Taken together, although *Helq^gt/gt^* mice exhibit hypogonadsim that is phenotypically similar to FA mouse models, its underlying mechanism is distinct, given the non-epistatic relationship between *Helq* and *Fancc.*

### *Helq^gt/gt^* mice are born in the expected Mendelian ratio, showing no growth retardation

As *Helq^gt/gt^* males are fertile, we performed crosses between *Helq^gt/gt^*;*Fancc^+/^*^−^ males and *Helq^gt/+^*;*Fancc^+/^*^−^ females to efficiently generate *Helq^gt/gt^*;*Fancc*^−^*^/^*^−^ mice in the B6 background. A total of 105 mice were genotyped at 3 weeks of age (Supplementary Table S2). Although *Helq^gt/gt^* mice were found at the expected ratio, the number of *Fancc*^−^*^/^*^−^ mice in this background was reduced to ∼65% of the expected number (17 versus 26.25, *P* < 0.05, χ^2^-test) as described previously (52). Only seven *Helq^gt/gt^*;*Fancc*^−^*^/^*^−^ mice were observed at this age, but this number was not statistically different from 13.125, the expected number (*P* > 0.05, χ^2^-test). This relatively small number of *Helq^gt/gt^*;*Fancc*^−^*^/^*^−^ mice is most likely attributed to the sub-lethality of *Fancc*^−^*^/^*^−^ mice in this background. As shown in Figure 2E, we also found that *Fancc*^−^*^/^*^−^ mice are significantly smaller in size (average weight of 7.4 g ± 0.61) compared with WT (8.7 ± 0.15 g) and *Helq^gt/gt^* mice (8.8 ± 0.39 g). *Helq^gt/gt^*;*Fancc*^−^*^/^*^−^ mice were even smaller than *Fancc*^−^*^/^*^−^ mice, weighing 6.0 g ± 0.71 on average, though this difference did not reach statistical significance likely due to the small number of mice examined. Overall, *Helq^gt/gt^* mice are healthy, exhibiting phenotypes milder than *Fancc*^−^*^/^*^−^ mice. It was reported that *Fancc*^−^*^/^*^−^ mice in the B6 background are essentially tumor-free (53). Similarly, a small-scale aging study with 11 *Helq^gt/gt^* mice (5 males and 6 females) in this background showed no significant increase in spontaneous tumor incidence up to 21 months of age. This was not surprising, given the milder phenotypes of *Helq^gt/gt^* mice compared with *Fancc*^−^*^/^*^−^ mice.

### Mono-ubiquitination and focus formation of FANCD2 are intact in *Helq^gt/gt^* cells

**Figure 3.**
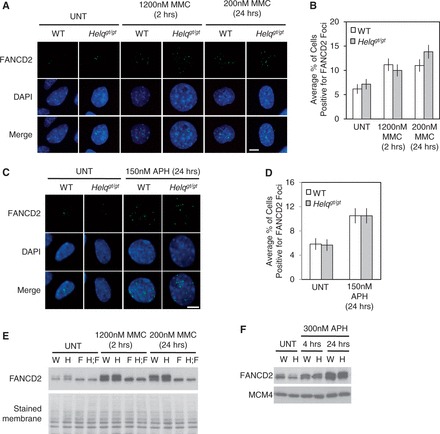
FANCD2 mono-ubiquitination and focus formation remain intact in *Helq^gt/gt^* cells. Shown are representative images of FANCD2 foci (green) in response to MMC (**A**) or APH (**C**) in WT and *Helq^gt/gt^* cells. Cells were harvested immediately following treatment except for the 2 h treatment of 1200 nM MMC, in which cells were given 22 h of recovery time before harvest. Nuclei were stained with DAPI (blue). Scale bar is 10 µm. The average percentages of cells positive for FANCD2 foci in response to MMC or APH are shown in (**B**) and (**D**), respectively. (**E**) Western blotting shows that *Helq^gt/gt^* cells exhibit normal FANCD2 mono-ubiquitination in response to MMC. The defect observed in *Fancc*^−^*^/^*^−^ and *Helq^gt/gt^;Fancc*^−^*^/^*^−^ cells is shown for a better comparison. (**F**) *Helq^gt/gt^* cells do not display any defect in FANCD2 mono-ubiquitination in response to APH. Cells were treated with 300 nM APH for either 4 or 24 h before harvest. Error bars (B, D) represent the binomial error for the combined data set. A stained membrane and MCM4 were used as loading controls in (E) and (F), respectively. W, H, F and H;F in (E, F) refer to WT, *Helq^gt/gt^*, *Fancc*^−^*^/^*^−^ and *Helq^gt/gt^;Fancc*^−^*^/^*^−^ cells, respectively. UNT refers to untreated cells.

Our data so far did not support epistasis between *Helq* and *Fancc.* Therefore, we next tested the role of *Helq* in FANCD2 focus formation, a signature of FA pathway activation (25). For this purpose, we used primary WT and *Helq^gt/gt^* MEFs, as *Fancc*^−^*^/^*^−^ and *Helq^gt/gt^*;*Fancc*^−^*^/^*^−^ cells, both lacking a functional FA core complex, do not form FANCD2 foci (25). As shown in Figure 3A and B, no significant difference was observed in the percentage of cells positive for FANCD2 foci between WT and *Helq^gt/gt^* cells even after treatment with the crosslinker MMC. The same was true after treatment with aphidicolin (APH), a replication inhibitor and robust inducer of FANCD2 and FANCI foci (54) (Figure 3C and D). Focus formation of FANCD2/FANCI at prophase, markers of unresolved replication intermediates (55), were also unchanged between these two genotypes in response to MMC or APH (Supplementary Figure S3A and B). Agreeing with these data, mono-ubiquitination of FANCD2 was also robustly induced in *Helq^gt/gt^* cells following treatment with either MMC or APH (Figures 3E and F). In comparison, no mono-ubiquitinated FANCD2 could be detected in *Fancc*^−^*^/^*^−^ and *Helq^gt/gt^*;*Fancc*^−^*^/^*^−^ cells. These data collectively suggest that *Helq* is not required for FANCD2 mono-ubiquitination or focus formation.

### HELQ plays a minor role in MMC resistance in a manner non-epistatic with FANCC

**Figure 4.**
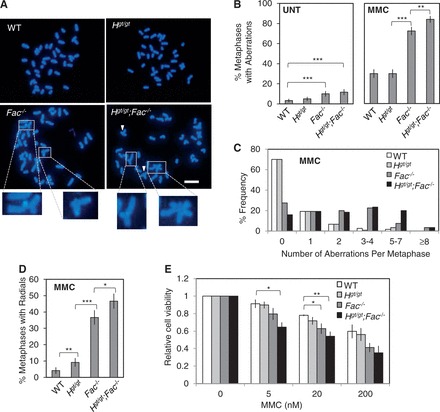
*Helq^gt/gt^* cells display modest sensitivity to MMC. (**A**) Shown are representative images of DAPI-stained metaphase spreads from all genotypes following MMC treatment (600 nM MMC for 2 h followed by 22 h recovery before harvest). White arrowheads indicate chromosome aberrations. Enlarged images of radial structures from the *Fancc*^−^*^/^*^−^ and *Helq^gt/gt^;Fancc*^−^*^/^*^−^ samples are shown at bottom. Scale bar is 10 µm. (**B**) Shown are the average percentages of metaphases positive for chromosomal aberrations in the untreated (left) or MMC-treated (right) conditions. For each experimental group, 120 metaphases were scored. (**C**) A histogram displaying the number of aberrations per metaphase for each genotype after MMC treatment is shown. (**D**) The average percentages of metaphases positive for radial structures after MMC treatment are shown. (**E**) The MTT assay reveals that *Helq^gt/gt^* cells display little, if any MMC sensitivity compared with *Fancc*^−^*^/^*^−^ or *Helq^gt/gt^;Fancc*^−^*^/^*^−^ cells. Cells were treated with the indicated doses of MMC for 5 days before analysis. Error bars show either the binomial error of the combined data set (B, D) or the SEMs for at least three independent experiments (E). Significance was determined by either χ^2^-test (B, D) or *t*-test (E). Statistical significance at *P* < 0.05, *P* < 0.01 and *P* < 0.001 is indicated as *, ** and ***, respectively. WT, *H^gt/gt^*, *Fac*^−^*^/^*^−^ and *H^gt/gt^;Fac*^−^*^/^*^−^ refer to WT, *Helq^gt/gt^*, *Fancc*^−^*^/^*^−^ and *Helq^gt/gt^;Fancc*^−^*^/^*^−^ respectively.

Previous studies reported that HELQ is required for ICL resistance in worms and humans (24,27) but not in chicken DT40 cells (14). Therefore, we tested *Helq^gt/gt^* cells for MMC hypersensitivity, another hallmark of FA cells (6). For this line of experiments, we used primary MEFs with the following four genotypes, WT, *Helq^gt/gt^*, *Fancc*^−^*^/^*^−^ and *Helq^gt/gt^;Fancc*^−^*^/^*^−^. First, we examined MMC-induced chromosome aberrations. Representative metaphase spreads for each genotype after MMC treatment are shown in Figure 4A. Although we scored 120 metaphases per experimental group, the total number of MMC-induced chromosome aberrations was not statistically different between *Helq^gt/gt^* and WT cells (Figure 4B and C). However, *Helq^gt/gt^* cells did exhibit a slight but significant increase in radials (9.2 ± 2.6% as opposed to 5.0 ± 2.0% in WT), a type of complex chromosome aberration that occurs frequently in MMC-treated FA cells (Figure 4A and D). Consistent with their hypersensitivity to MMC, the majority of *Fancc*^−^*^/^*^−^ cells exhibited chromosome aberrations (72.5 ± 4.09%, Figure 4B) with a drastic increase in radials (36.7 ± 4.42%, Figure 4D). Intriguingly, *Helq^gt/gt^;Fancc*^−^*^/^*^−^ cells showed statistically higher levels of chromosome aberrations including radials (84.5 ± 3.35% and 46.7 ± 4.57% in Figure 4B and D, respectively) compared with *Fancc*^−^*^/^*^−^ cells. These data suggest that *Helq^gt/gt^* cells are not as extremely sensitive to MMC as *Fancc*^−^*^/^*^−^ cells. However, as *Helq^gt/gt^;Fancc*^−^*^/^*^−^ cells showed a significantly higher number of MMC-induced chromosome aberrations than *Fancc*^−^*^/^*^−^ cells, HELQ contributes to MMC resistance through a mechanism that is distinct from the function of FANCC. Given the mild sensitivity of *Helq^gt/gt^* cells to MMC, this mechanism is likely to be a secondary alternative to the FA pathway. Finally, we tested the effect of MMC on the proliferation of these cells using a MTT assay. In agreement with the metaphase analysis, a 5 days culture in multiple low doses of MMC significantly reduced the proliferation of *Fancc*^−^*^/^*^−^ and *Helq^gt/gt^;Fancc*^−^*^/^*^−^, but not *Helq^gt/gt^*, cells (Figure 4E). Although *Helq^gt/gt^;Fancc*^−^*^/^*^−^ cells appeared to display an even greater reduction in proliferation than *Fancc*^−^*^/^*^−^ cells, this did not reach statistical significance. Together, these data are consistent with the idea of HELQ performing a minor role in MMC resistance.

### *HELQ* depletion leads to only mild MMC sensitivity compared with FANCA-depleted/FANCC-deficient human cells

Although *Helq^gt/gt^* cells apparently lack normal full-length HELQ, they do express the mutant HELQΔ-β-Geo protein. Therefore, we wondered whether the mild MMC sensitivity of *Helq^gt/gt^* cells truly reflects a consequence of HELQ deficiency. To test this possibility, we depleted *HELQ* and/or *FANCA*, another FA core complex member (56), in human HEK 293T cells via small interfering RNAs (siRNA, see Supplementary Figure S4A). At two different doses, *FANCA* depletion, but not *HELQ* depletion, led to MMC hypersensitivity as measured by colony formation assay (Supplementary Figure S4B). Similar results were obtained for MMC-induced chromosome aberrations (Supplementary Figures S4C and D). To further confirm these results, we also performed siRNA-mediated depletion of *HELQ* in PD331 cells (Supplementary Figure S4E), a human cell line deficient for *FANCC*, or their complemented counterparts (PD331 + FANCC). In a colony formation assay, *HELQ*-depleted PD331 + FANCC cells exhibited modestly decreased survival following MMC treatment (at 300 nM) compared with control siRNA-treated counterparts, though this was still mild compared with that of the PD331 cells (Supplementary Figure S4F). Only when measured by MTT assay (5 days culture, 15–45 nM doses) could we observe a clear non-epistatic relationship between *HELQ* and *FANCC* (Supplementary Figure S4G). Together, these data collectively support the idea that HELQ plays a minor, backup role in MMC resistance in mammalian cells that is most likely non-epistatic to *FANCC*.

### A loss of HELQ and/or FANCC alters the distribution of replication fork speed in unperturbed S phase, increasing persistent stalled forks

**Figure 5.**
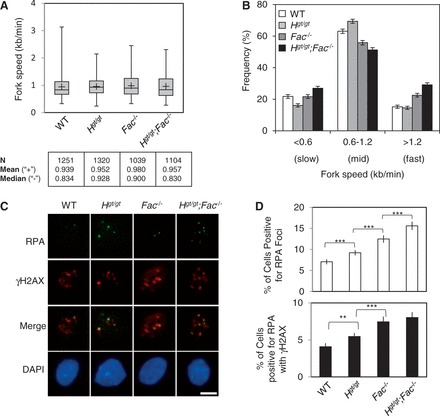
A combined loss of *Helq* and *Fancc* greatly alters the distribution of replication fork speeds and leads to increased levels of RPA/γH2AX foci. (**A**) Box plots show the range of fork speed values for the four genotypes. The line through the middle of the shaded box represents the median, whereas the ‘+’ sign shows the location of the mean (values shown at bottom along with the number of tracts, N, analyzed). (**B**) Separating fork speed values into slow, mid and fast forks reveals statistically significant differences among the ratios of the four genotypes (*P* < 0.001, χ^2^-test). Error bars show the binomial error. (**C**) Shown are representative images of cells from all four genotypes co-stained for RPA (green) and γH2AX (red). Nuclei were stained with DAPI (blue). For RPA foci analysis, cells were pre-extracted before fixation using a 0.5% Triton X-100 solution. Scale bar is 10 µm. (**D**) The average percentages of cells positive for RPA foci (top) or RPA/γH2AX co-localization events (bottom) are shown. Error bars show the binomial error for the combined data set. Statistical significance (determined by χ^2^-test) at *P* < 0.01 and *P* < 0.001 are indicated as ** and ***, respectively. WT, *H^gt/gt^*, *Fac*^−^*^/^*^−^ and *H^gt/gt^;Fac*^−^*^/^*^−^ refer to WT, *Helq^gt/gt^*, *Fancc*^−^*^/^*^−^ and *Helq^gt/gt^;Fancc*^−^*^/^*^−^, respectively.

Several studies have suggested that HELQ and its orthologs have a role in the recovery of stalled or collapsed replication forks (23,38,57). As we noticed a modest but significant increase in spontaneous chromosome aberrations in *Fancc*^−^*^/^*^−^ and *Helq^gt/gt^;Fancc*^−^*^/^*^−^ cells (Figure 4B), we examined replication fork speed in WT, *Helq^gt/gt^*, *Fancc*^−^*^/^*^−^ and *Helq^gt/gt^;Fancc*^−^*^/^*^−^ cells in unperturbed S phase using the DNA fiber technique (Supplementary Figure S5A) (29). Although the mean fork speeds were not different among the four genotypes (Figure 5A), the distributions of fork speed values were significantly different (*P* < 0.001, Kolmogorov–Smirnov test, Supplementary Figure S5B). Categorizing fork speeds into three ranges (slow, mid and fast), we found that compared with WT cells, *Helq^gt/gt^* cells exhibited a slight increase of forks in mid-range speed and a decrease in slow forks (Figure 5B). *Fancc*^−^*^/^*^−^ cells had a higher frequency of faster forks with a decrease of mid-speed forks. Increases in mid-speed and faster forks in these cells might have contributed to faster median fork speeds in these cells (Figure 5A). *Helq^gt/gt^;Fancc*^−^*^/^*^−^ cells displayed increases in both faster and slower fork speeds, indicating that their fork movement is greatly altered from WT cells even in unchallenged S phase. Although counterintuitive, an increase in faster forks may suggest an increase in fork stalling events, as we previously reported (29). This is due to the number of fork termination events. Faster forks that terminate within the duration of the assay likely escape detection. Thus, an increase in fork stalling leads to a decreased number of fork termination events, thereby leaving a greater number of faster forks visible for measurement. To address whether this was the case, we next looked at RPA32 foci, markers of stalled replication forks (Figure 5C) (58,59). Compared with WT cells (7.06 ± 0.61%), increased percentages of *Helq^gt/gt^* and *Fancc*^−^*^/^*^−^ cells (9.20 ± 0.61% and 12.5 ± 0.88%, respectively) were positive for RPA32 foci (Figure 5D), with an even further increase observed in *Helq^gt/gt^;Fancc*^−^*^/^*^−^ cells (15.6 ± 0.98%). These differences were all significant to one another (*P* < 0.001, χ^2^-test). The number of RPA32 foci co-localizing with γH2AX foci, a marker of DSBs (60), was also significantly increased in *Helq^gt/gt^* and *Fancc*^−^*^/^*^−^ cells (5.46 ± 0.48% and 7.44 ± 0.70%, respectively) compared with WT cells (4.08 ± 0.47%). However, unlike RPA32 foci, there was no significant increase in RPA/γH2AX co-localizing foci in *Helq^gt/gt^;Fancc*^−^*^/^*^−^ cells (8.02 ± 0.73%) compared with *Fancc*^−^*^/^*^−^ cells. These data present a new line of evidence that *Helq* and *Fancc* are not epistatic to each other in the recovery of stalled/collapsed replication forks even in unchallenged S phase.

### *Helq* and *Fancc* are independently required to prevent the formation of spontaneous MN

**Figure 6.**
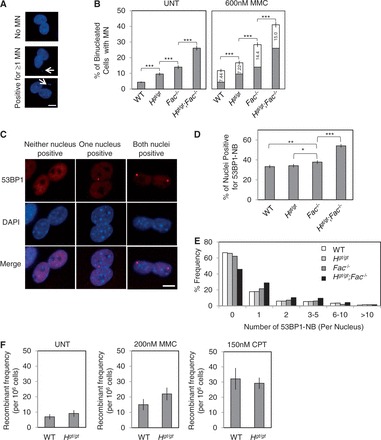
*Helq* suppresses multiple forms of spontaneous genome instability in a manner that is not epistatic with *Fancc*, but *Helq^gt/gt^* cells show levels of recombination comparable with WT. (**A**) Shown are representative images of binucleated cells with or without micronuclei (MN, indicated by white arrows). Nuclei and MN were stained with DAPI (blue). Scale bar is 10 µm. (**B**) Shown are the average percentages of binucleated cells positive for spontaneous MN (left) or MMC-induced MN (right). For the latter, the levels for the untreated condition are shown in gray with numbers in the white box showing the increase after MMC treatment. At least three independent experiments were performed and >900 cells were observed per experimental group. (**C**) Simultaneous disruption of *Helq* and *Fancc* results in a significant increase in 53BP1-NB. Shown are representative images of binucleated cells with or without nuclei positive for 53BP1-NB (red). Nuclei were stained with DAPI (blue). Scale bar is 10 µm. (**D**) Shown are the average percentages of nuclei positive for 53BP1-NB. (**E**) A histogram detailing the number of 53BP1-NB per nucleus is shown. Four independent experiments were performed and >750 cells were observed per experimental group. (**F**) No significant difference in the number of HR events was detected between WT and *Helq^gt/gt^* MEFs as measured by the *FYDR* transgenic locus system. Shown are the average numbers of eYFP^+^ recombinants per 10^6^ cells analyzed. Scale bars in (B, D) show the binomial error of the combined data sets, wherease those in (F) show the SEMs for data obtained from at least seven different embryos per genotype. Statistical significance was determined by either χ^2^-test (B, D) or *t*-test (F). *P* < 0.05, *P* < 0.01 and *P* < 0.001 are indicated as*, ** and ***, respectively. WT, *H^gt/gt^*, *Fac*^−^*^/^*^−^ and *H^gt/gt^;Fac*^−^*^/^*^−^ refer to WT, *Helq^gt/gt^*, *Fancc*^−^*^/^*^−^ and *Helq^gt/gt^;Fancc*^−^*^/^*^−^, respectively.

Persistent stalled forks can lead to the formation of micronuclei (MN) if unresolved before M phase entry (29,61). Therefore, we measured spontaneous MN levels using the cytokinesis-block MN assay (Figure 6A), a standard assay for this purpose (31). Compared with WT cells (4.33 ± 0.48%, see Figure 6B), significantly increased numbers of *Helq^gt/gt^* (9.56 ± 0.98%) and *Fancc*^−^*^/^*^−^ (14.0 ± 1.16%) cells contained spontaneous MN (*P* < 0.001, χ^2^-test for both). *Helq^gt/gt^;Fancc*^−^*^/^*^−^ cells exhibited a >6-fold increase (26.1 ± 1.47%) compared with WT cells, and this number was also statistically higher than that of *Fancc*^−^*^/^*^−^ cells (*P* < 0.001, χ^2^-test). These data indicate that *Helq* and *Fancc* function in a non-epistatic manner to prevent spontaneous MN likely through contributing to the recovery of stalled forks. We exploited this modest but significant increase in spontaneous MN in *Helq^gt/gt^* cells to validate that this allele accurately reflects the consequences of loss of HELQ function. We performed siRNA-mediated knockdown of *Helq* (or *Helq^gt^*) transcripts in WT and *Helq^gt/gt^* MEFs, respectively (Supplementary Figures S6A and B). This resulted in significantly increased spontaneous MN levels in WT cells (*P* < 0.001, χ^2^-test) but not *Helq^gt/gt^* cells (Supplementary Figure S6C), suggesting that the HELQΔ-β-Geo mutant protein is likely devoid of any activity. Unresolved replication intermediates can cause the formation of MN through two mechanisms; non-disjunction of sister chromatids and chromosome/chromatid breaks. These two mechanisms can be distinguished by staining MN for the centromeric marker CENP-A (62) (Supplementary Figure S6D). We found that both types of MN were increased in these mutant cells with similar ratios (Supplementary Figure S6E). We also measured MMC-induced MN in these cells. Because of the relatively higher levels of spontaneous MN in mutant cells, we subtracted spontaneous MN frequency values from those in the MMC treatment to obtained differences (numbers in the white bars in Figure 6B). These were then compared to evaluate the effect of MMC on MN formation. Such values were similar between WT and *Helq^gt/gt^* cells (7.44 and 7.22, respectively), whereas *Fancc*^−^*^/^*^−^ cells showed a 2-fold larger value (14.1) at the same dose used for the metaphase analysis (Figure 4). These findings are consistent with the idea that the FA pathway is a major pathway for MMC resistance for which HELQ performs only a minor role. *Helq^gt/gt^;Fancc*^−^*^/^*^−^ cells showed a value (15.0) that was not much higher than that of *Fancc*^−^*^/^*^−^ cells. We think this may be due to the extreme sensitivity of *Helq^gt/gt^;Fancc*^−^*^/^*^−^ cells to MMC. As the majority of them have multiple abnormal metaphase chromosomes (Figure 4C), a fraction of them may not complete M phase to form MN in the subsequent G1 phase, making this value smaller. Unlike spontaneous conditions, MMC increased exclusively CENP-A- MN (Supplementary Figure S6E). We also examined CPT-induced MN (Supplementary Figure S6F). Both *Helq^gt/gt^* and *Fancc*^−^*^/^*^−^ cells showed significantly increased levels of CPT-induced MN (14.2 and 14.2, respectively) compared with WT cells (9.93). The largest increase was observed in *Helq^gt/gt^;Fancc*^−^*^/^*^−^ cells (16.2). However, we were unable to determine epistasis for this, due to the limited sensitivity of the MN assay.

### *Helq* and *Fancc* are not epistatic to suppress the formation of 53BP1 nuclear bodies

Recently, it was shown that unresolved replication intermediates can rupture during passage through M phase, leading to the formation of what are known as 53BP1 nuclear bodies (53BP1-NB) in G1 phase cells (63,64). Interestingly, such bodies are often exquisitely symmetrical in terms of their appearance within the daughter nuclei (Figure 6C). To score 53BP1-NB, G1 phase cells are typically identified as cyclin A negative (63,64). However, owing to the lack of a cyclin A antibody that works well for mouse cells, we used the cytokinesis-blocking reagent, cytochalasin B, to identify G1 phase daughter nuclei as those contained within binucleated cells (Figure 6C). Although there was no significant difference in the percentage of 53BP1-NB positive nuclei between WT and *Helq^gt/gt^* cells (33.3 ± 1.67% and 34.2 ± 1.68%, respectively), the number of nuclei containing 53BP1-NB was statistically higher in *Fancc*^−^*^/^*^−^ cells (37.8 ± 1.71%, *P* < 0.01, χ^2^ test) (Figure 6D). Much like spontaneous MN formation (Figure 6B), *Helq^gt/gt^;Fancc*^−^*^/^*^−^ cells showed a drastic increase in 53BP1-NB containing nuclei (54.1 ± 1.77%) compared with *Fancc*^−^*^/^*^−^ cells (*P* < 0.001, χ^2^ test) with more nuclei containing multiple 53BP1-NB (Figure 6E). These data are consistent with the non-epistatic relationship between *Helq* and *Fancc* in preventing genome instability derived from persistent stalled forks.

### *Helq^gt/gt^* cells display recombinant frequencies at the *FYDR* locus that are comparable with WT

Given its involvement in meiotic DSB repair in flies and worms (20,21), we postulated that HELQ may function downstream of the FA pathway in HR, similar to other proteins such as BRCA2 and PALB2 (65,66). Supporting this idea, it has been reported that depletion of *HELQ* in human cells lowers HR efficiency (27). To test this, we used the *FYDR* transgenic locus system (32) to measure the levels of spontaneous, MMC-induced and CPT-induced HR events in *Helq^gt/gt^* MEFs (Supplementary Figures S7A and B). Under all conditions tested, *Helq^gt/gt^* cells showed levels of recombination that were comparable with WT (Figure 6F and Supplementary Figure S7C), suggesting the possibility that HELQ is not a major player in HR.

## DISCUSSION

In this study, we have investigated a possible involvement of HELQ in the FA pathway using the *Helq^gt^* strain as a model. We showed that primary *Helq^gt/gt^* MEFs are capable of FANCD2 mono-ubiquitination and focus formation (Figure 3). By phenotypic comparison to *Fancc*^−^*^/^*^−^ mice/cells in the same genetic background, we found that *Helq^gt/gt^* mice/cells exhibit a mild form of FA-like phenotypes such as hypogonadism (Figure 2) and MMC sensitivity (Figure 4). Importantly, double mutants for *Helq^gt^* and *Fancc*^−^ had more severe phenotypes than single mutants (Table 1). These findings are in stark contrast to the complete epistasis reported for *Fanca/Fancc* and *Fanca/Fancg* in mice (50,67). Collectively, our data show that *Helq* and *Fancc* are not epistatic to one another for any trait tested.

**Table 1.** Summary of phenotypes following *Helq* and/or *Fancc* disruption

Trait	WT	*Helq^gt/gt^*	*Fancc*^−^*^/^*^−^	*Helq^gt/gt^; Fancc*^−^*^/^*^−^
Sub-lethality	−	−	+	+
Growth retardation	−	−	+	++?
Tumor	−	−	−^(52)^	N.D.
Hypogonadism	−	+	++	+++
MMC sensitivity	−	+/−	++	+++
MN formation	−	+	++	+++
53BP1-NB formation	−	−	+	++
HR (measured by *FYDR*)	−	−	N.D.	N.D.

‘−’ indicates no significant change from WT, whereas ‘+’, ‘++’ and ‘+++’ refer to progressively more severe phenotypes. ‘+/−’ refers to a small but significant effect. N.D. means not determined.

Although our data strongly indicate that HELQ and FANCC function in parallel, it remains possible that HELQ could function in HR as a downstream step in the FA pathway. If this is the case, then the non-epistatic relationship between *Helq* and *Fancc* implicates that the FA core complex and HR machinery have a complicated non-linear relationship as seen previously for *fancc* and *brca2* in chicken DT40 cells (68). However, using the *FYDR* transgenic locus system, we found that HELQ’s role in HR is likely to be non-essential or minor (Figure 6F). This is further supported by the fact that (i) *Helq^gt/gt^* mice are fully viable as opposed to early embryonic lethality seen for disruption of major HR genes (69), (ii) loss of HELQ results in only modest sensitivity to MMC (Figure 4), and (iii) *Helq^gt/gt^* males are fertile, showing no apparent meiosis defects. Alternatively, it may be that the role of HELQ in HR is only visible when a major player in HR is compromised. Thus, the precise role of HELQ in HR requires further investigation.

Our data show that loss of *Helq* in mice results in phenotypes considerably milder compared with those seen in *Fancc*^−^*^/^*^−^ mice (Table 1). This observation was not limited to *Helq^gt^* homozygosity, as *HELQ* depletion in human HEK 293T cells did not cause a statistically higher level of MMC-induced chromosome aberrations or affect cellular survival as measured by colony formation assay (Supplementary Figure S4A–D). Furthermore, *HELQ*-depleted PD331 + FANCC cells exhibited only modestly reduced cellular survival at 300 nM MMC (colony formation assay) (Supplementary Figure S4F). Although our data show a consistent trend towards HELQ and FANCA/FANCC being non-epistatic in human cells, the minor role of HELQ in MMC resistance may have prevented it from being manifested as statistically significant, except in the MTT assay (Supplementary Figure S4G).

It is noteworthy that HELQ orthologs do not exist in bacteria or yeast but archaea have HELQ-like helicases (HELQa) (70). Atomic structures of HELQa from three species revealed the presence of five structural domains in HELQ, which are also conserved in HELQ in metazoans (36,38,71). Mutagenesis studies have demonstrated that normal helicase activity requires the three C-terminal domains (36,72), which are missing from HELQΔ-β-Geo in *Helq^gt/gt^* mice (Figure 1B). Therefore, it is likely that HELQΔ-β-Geo is also devoid of helicase activity. Furthermore, the presence of β-Geo is also likely to jeopardize its enzymatic activity. In line with this, siRNA-mediated knockdown of the *Helq^gt^* transcript in *Helq^gt/gt^* cells did not lead to increased levels of spontaneous MN (Supplementary Figure S6C). Furthermore, given the normal phenotypes of *Helq^gt/+^* mice, we do not think that this allele confers any dominant-negative effect. Future studies using a null allele may give definitive answers for these issues.

Although there are definitely certain differences, HELQa and mammalian HELQ share similar biochemical properties with preference for structures resembling stalled replication forks, suggesting their role in stalled fork recovery (23,57,73). Consistent with this idea, loss of HELQ caused an increase in stalled forks even in unchallenged conditions, and this role of *Helq* was not epistatic to *Fancc* (Figure 5D). The non-epistatic relationship for *Helq* and *Fancc* is much clearer for the formation of spontaneous MN and 53BP1-NB (Figure 6), which are derived from persistent stalled forks, rather than MMC-induced chromosome aberrations (Figure 4). Therefore, we propose that the major role of HELQ is the rescue of stalled forks in normal S phase. Given that HELQ and the FA core complex function in parallel, elucidating such a role of HELQ may provide clues to decipher the function of the FA pathway in physiological conditions beyond ICL repair. Furthermore, as HELQ remains functional in FA mutant cells, it could potentially be exploited to provide a therapeutic benefit against cancers with FA pathway disruption.

Recent genome-wide association studies have identified single nucleotide polymorphisms at loci within or near *HELQ* that are associated with increased risks for several different cancers including upper aerodigestive tract cancers and head and neck cancers (74–77). Our study was unable to detect an increased incidence of spontaneous tumors in *Helq^gt/gt^* mice. This could be due to several different factors, such as genetic background and species difference. The majority of FA mouse models do not show a strong cancer phenotype, despite the FA pathway’s tumor suppressive role in humans (39). Therefore, it may be necessary to test the role of *Helq* in tumor suppression in a sensitized background.

## SUPPLEMENTARY DATA

Supplementary Data are available at NAR Online.

## FUNDING

A postdoctoral fellowship from the Japan Society for the promotion of Science (to T.K.); March of Dimes [5-FY07-110 to N.S.]; and National Institutes of Health (NIH) [R01CA148806 to N.S.]. Funding for open access charge: University of Minnesota.

*Conflict of interest statement.* None declared.

## Supplementary Material

Supplementary Data
