# Supplementary material for: Helq acts in parallel to Fancc to suppress replication-associated genome instability
Source: Nucleic Acids Res. 2013 Aug 21;41(22):10283–97. doi: 10.1093/nar/gkt676 (PMC3905894; doi:10.1093/nar/gkt676)
Supplement: Supplementary Data [file supp_41_22_10283__index.html]

Helq acts in parallel to Fancc to suppress replication-associated genome instability — Helq acts in parallel to Fancc to suppress replication-associated genome instability — Supplementary Data 

# *Helq* acts in parallel to *Fancc* to suppress replication-associated genome instability

## 

files

**Files in this Data Supplement:**

- Supplementary Data - pdf file
