# Supplementary material for: Helq acts in parallel to Fancc to suppress replication-associated genome instability
Source: Nucleic Acids Res. 2013 Aug 21;41(22):10283–97. doi: 10.1093/nar/gkt676 (PMC3905894; doi:10.1093/nar/gkt676)
Supplement: Supplementary Data [file supp_gkt676_nar-01196-d-2013-File009.pdf]

Supplementary Figure S1. Luebben et al.

A

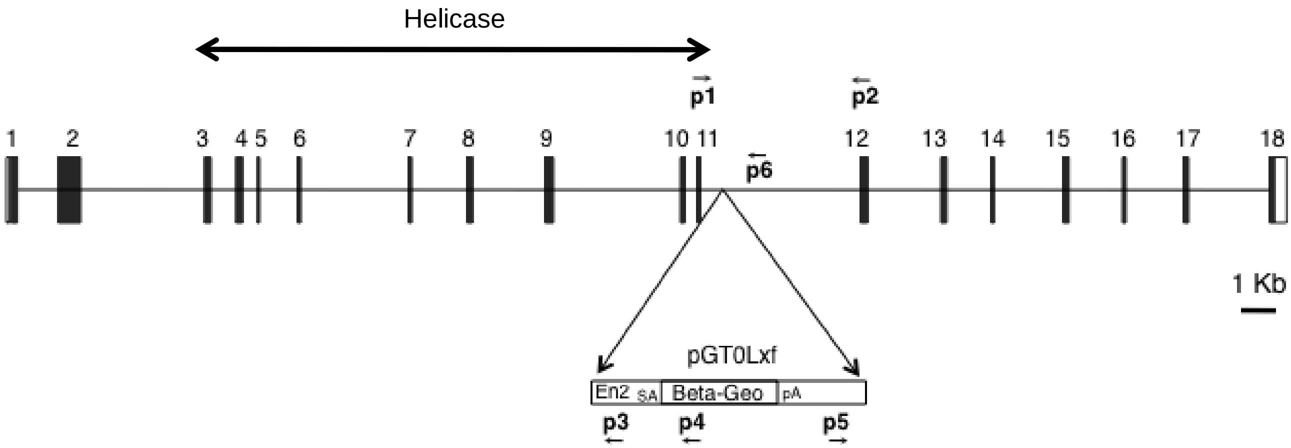

B Genomic PCR

Beginning of the insertion
(Primers p1 + p3)

End of the insertion
(Primers p5 + p6)

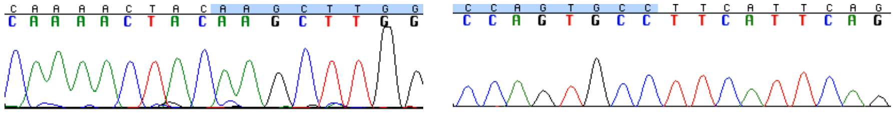

C RT-PCR

Exons 11-12 transcript
(Primers p1 + p2)

Chimeric transcript
(Primers p1 + p4)

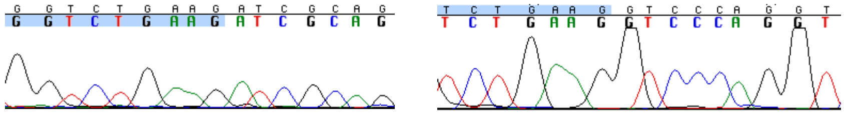

Supplementary Figure S2. Luebben et al.

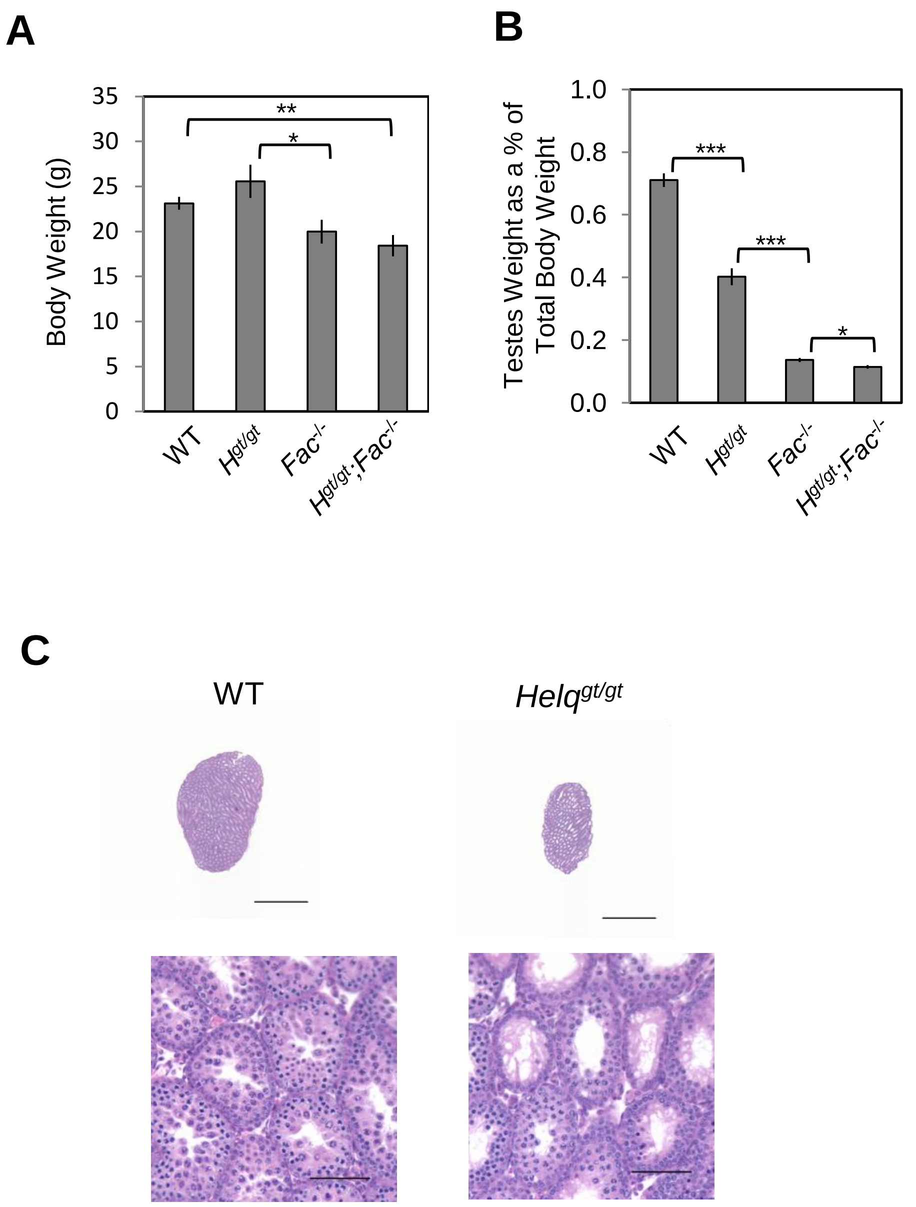

Supplementary Figure S3. Luebben et al.

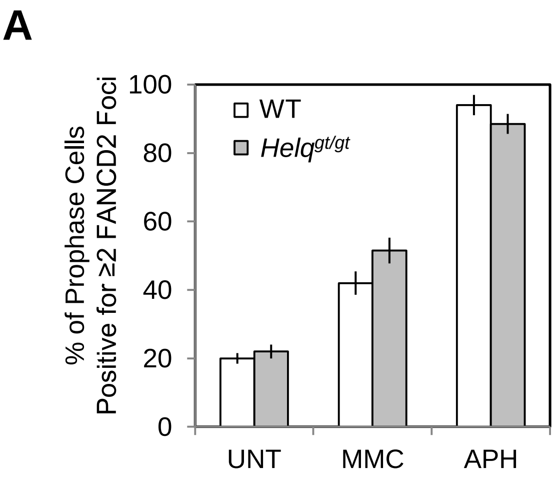

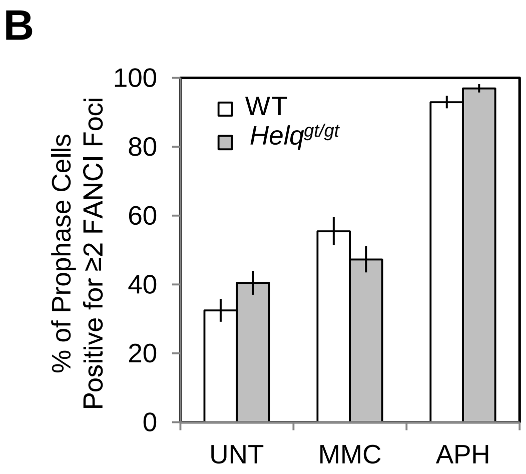

Supplementary Figure S4. Luebben et al.

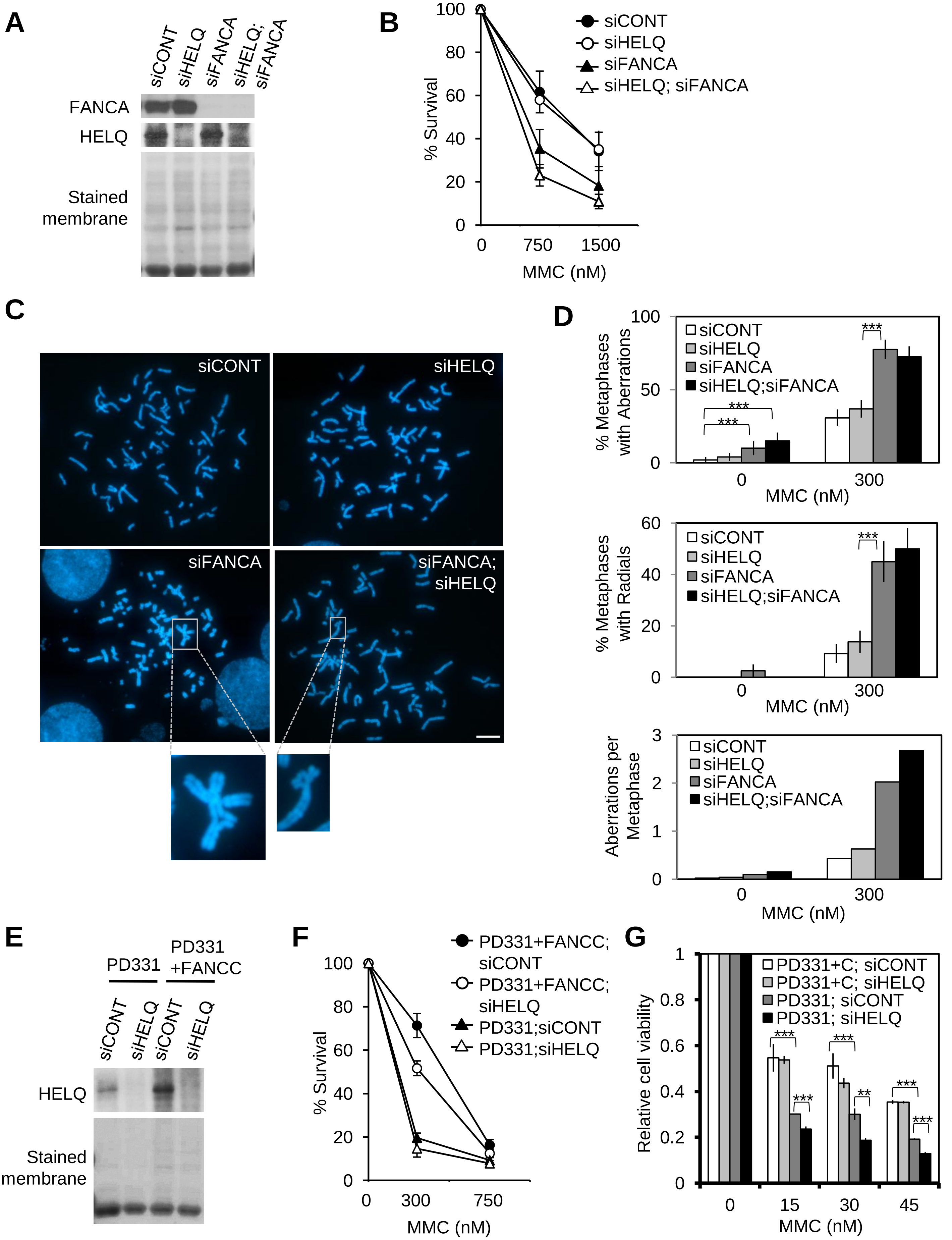

Supplementary Figure S5. Luebben et al.

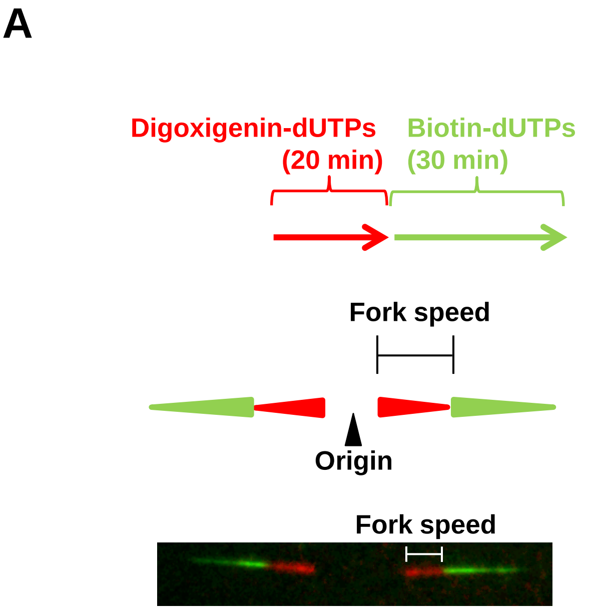

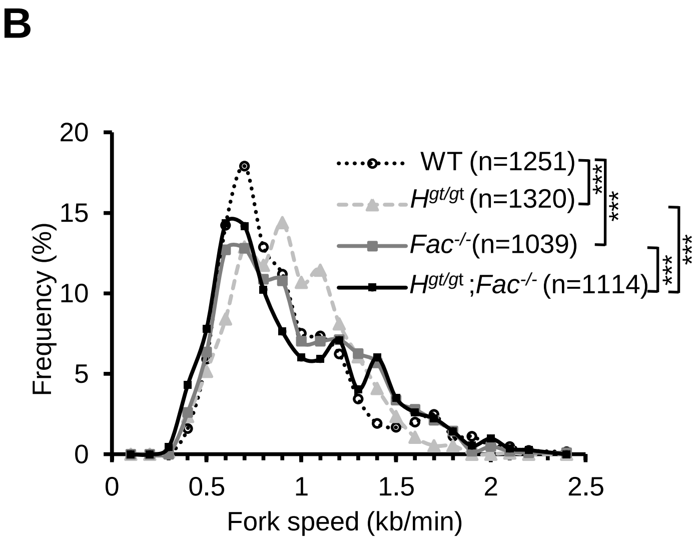

Supplementary Figure S6. Luebben et al.

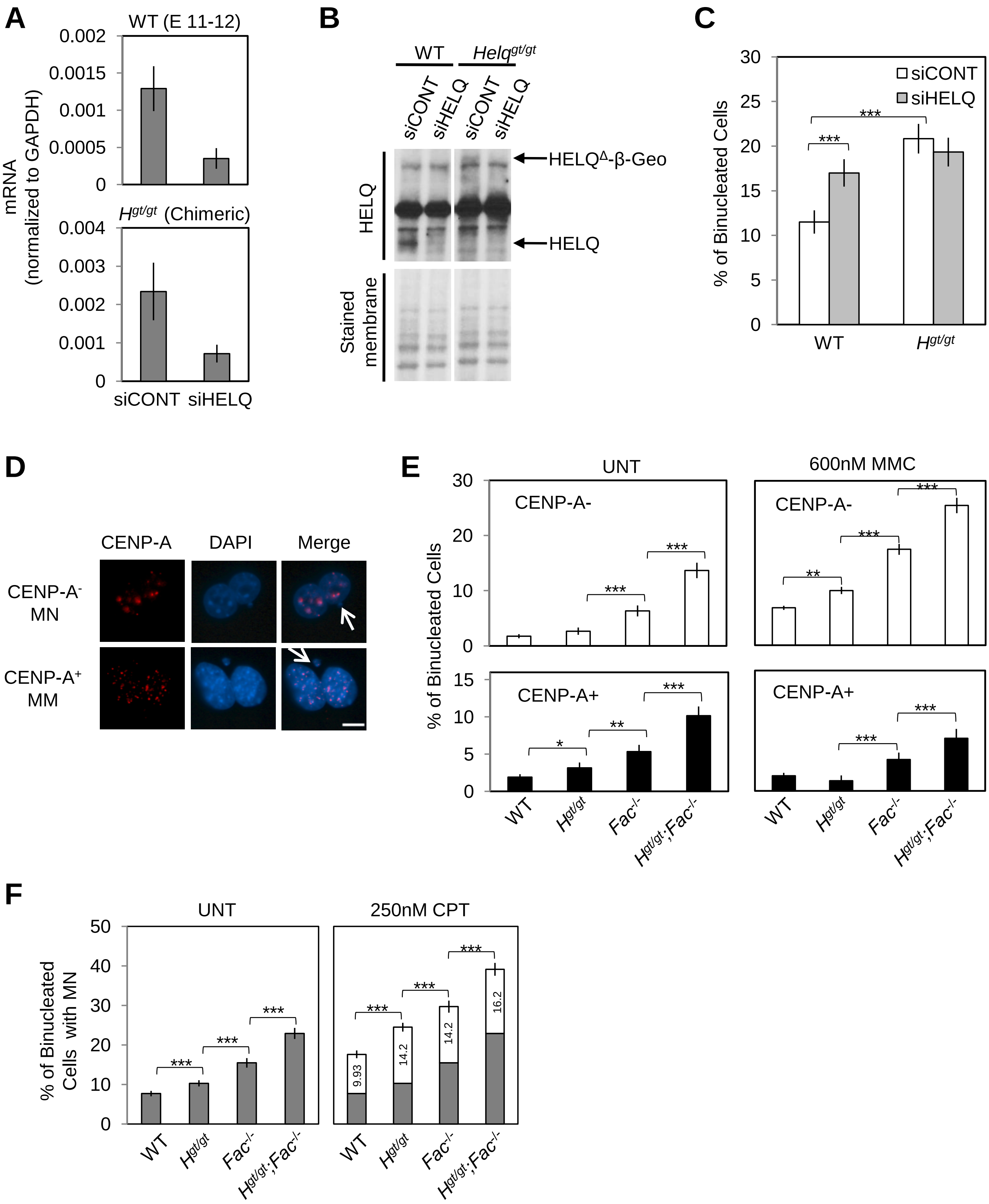

Supplementary Figure S7. Luebben et al.

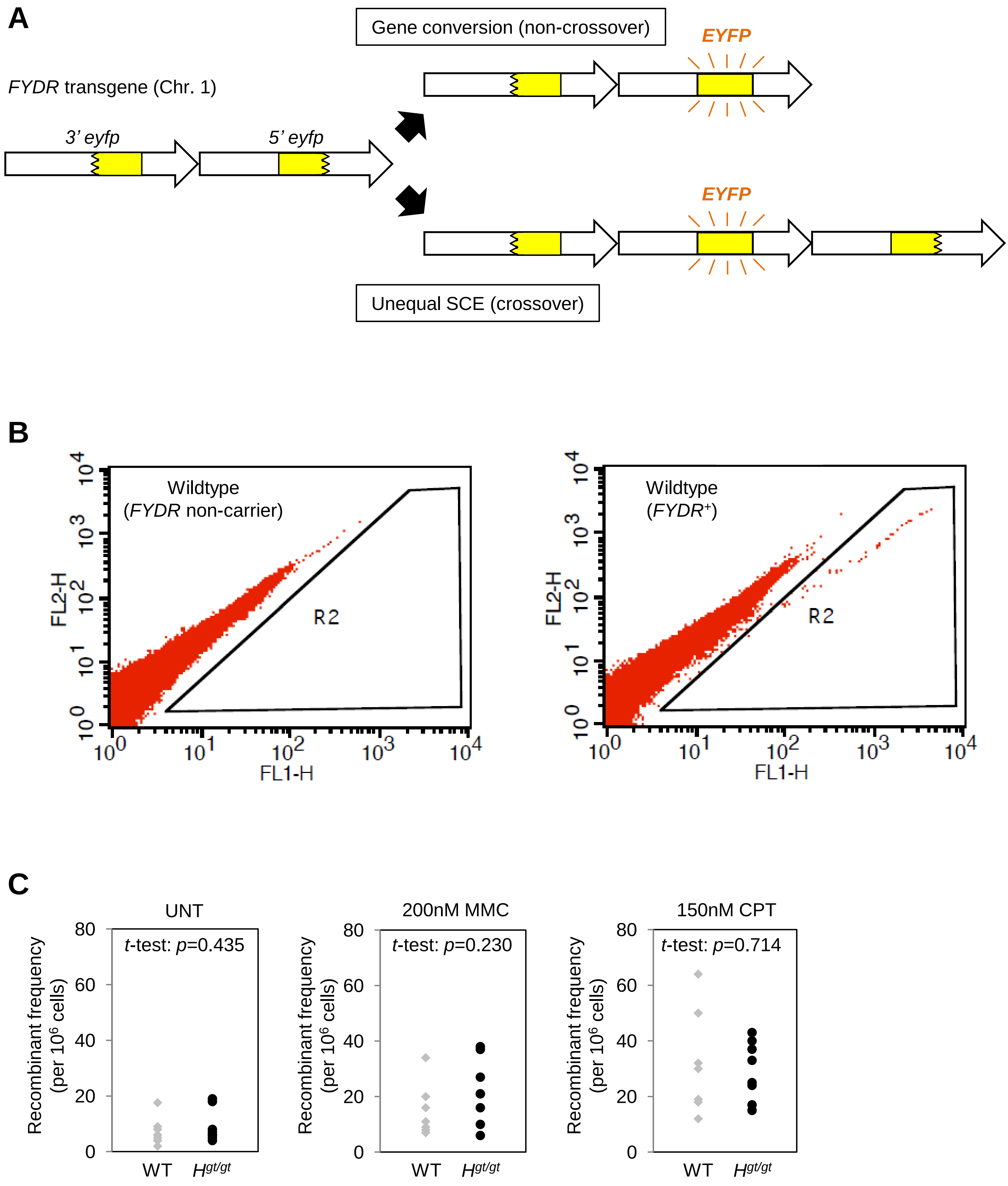

Supplementary Figure Legends

Supplementary Figure S1. Structure of the *Helq*^{gt} allele. (A) A diagram (drawn to scale) depicting the insertion of the gene-trap vector (pGT0Lxf) into the mouse *Helq* locus is shown. Exons are shown as rectangles and the coding region is filled with black. The region corresponding to the DEAD/DEAH box is indicated by the thick double-sided arrow. The gene-trap vector consists of the En2 (engrail 2) intron and the subsequent splice acceptor site (SA), as well as a β-Geo reporter gene followed by a polyadenylation signal (pA). (B) PCR using genomic DNA from *Helq*$^{gt/gt}$ mice reveals the insertion of the gene-trap vector (8.6kb) within the intron between exons 11-12. Sequencing chromatograms are shown with the shaded area highlighting sequence from the vector. Primer pairs p1 & p3 and p5 & p6 indicated in (A) were used to map the beginning and end of the insertion, respectively. Right after the end of the vector insertion, a 13-bp sequence in the intron was replaced with an 8-bp sequence of unknown origin. (C) RT-PCR on total RNA from testes shows the presence of the wildtype (with exons 11 and 12) and chimeric transcripts. The sequencing chromatogram on the left is sequence from the wildtype transcript showing the junction between exons 11 and 12 (amplified with primers p1 and p2). On the right is the sequence of the junction between exon 11 and the vector amplified using primers p1 and p4. Shaded areas indicate the end of the exon 11 sequence. Information on all primers is available upon request.

Supplementary Figure S2. *Helq*$^{gt/gt}$ mice are normal in size but display a significant reduction in testes size. (A) Shown are the average body weights of mice from the four genotypes at the age of testes analysis (6 weeks of age). (B) Shown is the average testes weight as a percentage of total body weight for the four genotypes. Bars in (A) and (B) show the SEMs for ≥ 5 mice. Statistical significance (determined by t-test) at p<0.05, p<0.01 and p<0.001 are indicated as *, ** and **, respectively. WT, *H*$^{gt/gt}$, *Fac*$^{-/-}$, *H*$^{gt/gt}$;*Fac*$^{-/-}$ refers to wildtype, *Helq*$^{gt/gt}$, *Fancc*$^{-/-}$, *Helq*$^{gt/gt}$;*Fancc*$^{-/-}$, respectively. (C) The mosaic pattern of seminiferous tubules is more apparent in testes of *Helq*$^{gt/gt}$ mice at 3 weeks of age. Shown are H&E images of whole testis (top) and seminiferous tubules (bottom) from widltype (WT) and *Helq*$^{gt/gt}$ mice. Scale bars are 1500 μm for the whole testis sections and 75 μm for the enlarged images.

Supplementary Figure S3. *Helq*$^{gt/gt}$ cells show normal levels of FANCD2 and FANCI focus formation in prophase. Shown are the average percentages of cells positive for ≥2 FANCD2 (A) or FANCI (B) foci at prophase. Prophase cells were identified as those which displayed bright staining for phospho-Histone H3 (Ser10) and had not yet proceeded to prometaphase. APH treatment was 150 nM for 24 hrs. MMC treatment was 1.2 μM for 2 hrs followed by 22-hr recovery in fresh media. Experiments were repeated twice with different MEF lines. At least 140 prophases were scored per experimental group. Bars show the binomial error for the combined data set.

Supplementary Figure S4. *HELQ* depletion causes only mild MMC sensitivity compared to FA core complex-depletion/deficiency in human cell lines. (A) Western blotting shows that siRNA pools targeting HELQ (siHELQ) and/or FANCA (siFANCA) efficiently deplete HELQ and FANCA, respectively, to sub-detectable levels in HEK 293T cells, while a control siRNA pool (siCONT) has no effect. (B) Depletion of HELQ does not confer MMC hypersensitivity in HEK 293T cells. Shown are the results of two independently performed colony formation assays at the indicated doses of MMC. Treatment with MMC was for 2 hours, followed by a one-week culture period. (C) Shown are representative images of metaphase spreads from HEK 293T cells following 24 hours treatment with 300nM MMC. Enlarged images show representative radial structures observed in the siFANCA (bottom left) and siHELQ;siFANCA (bottom right) samples. Scale bar is 10 µm. (D) FANCA depletion, but not HELQ depletion, leads to a statistically significant increase in metaphase chromosomal aberrations in response to MMC. Shown are the average percentages of metaphases positive for chromosomal aberrations (top) or radial structures (middle). The average number of aberrations per metaphase is shown at bottom. At least 40 metaphases were scored per experimental group. (E) siHELQ efficiently depletes HELQ to sub-detectable levels in the PD331 (or PD331+FANCC complemented) cell lines, while siCONT has no effect. (F) Depletion of HELQ confers modest MMC hypersensitivity in the PD331+FANCC cell line. Shown are the results of two independently performed colony formation assays at the indicated doses of MMC. Treatment with MMC was for 2 hours, followed by a two-week culture period. (G) An MTT assay reveals that HELQ depletion further decreases the proliferation of PD331 (*FANCC*-deficient) cells, suggesting non-epistasis between *HELQ* and *FANCC*. MMC treatment was for 5 days at the indicated doses. A stained membrane

was used as a loading control in (A,E). Error bars in (B, F, G) show the SEMs for at least three independent experiments while those in (D) indicate the binomial error. Statistical significance was determined by either t-test (B, F, G) or χ^2-test (D). Significance at p<0.01 and p<0.001 are indicated as ** and ***, respectively.

Supplementary Figure S5. (A) A diagram depicting the DNA fiber assay is shown. In this assay, ongoing replication forks are observed via the sequential incorporation of digoxigenin- (red) or biotin (green)-conjugated dUTPs. Fork speed measurements are then made by measuring the distance between the start of the red tract to the start of the green tract. (B) The distributions of fork speed values for the four genotypes are shown. Slightly different patterns between the four genotypes result in statistically significant differences as measured by Kolmogorov-Smirnov test. (p<0.001 indicated as ***). WT, $H^{gt/gt}$, $Fac^{-/-}$, $H^{gt/gt};Fac^{-/-}$ refers to wildtype, $Helq^{gt/gt}$, $Fancc^{-/-}$, $Helq^{gt/gt};Fancc^{-/-}$, respectively.

Supplementary Figure S6. (A) qRT-PCR analysis using total RNA reveals that siHELQ was able to efficiently deplete both the wildtype *Helq* transcript (E 11-12, top) in wildtype MEFs as well as the chimeric mutant transcript (Chimeric, bottom) in $Helq^{gt/gt}$ MEFs by ~70% compared to siCONT-treated cells. Experiments were duplicated using RNA samples from different MEF lines to confirm reproducibility. A representative qRT-PCR data set is shown. (B) Western blotting shows that siHELQ depletes HELQ or HELQ$^{\Delta}$-β-Geo to sub-detectable levels in wildtype or $Helq^{gt/gt}$ MEFs, respectively. A stained membrane was used as a loading control. (C) Depletion of HELQ$^{\Delta}$-β-Geo does not have any effect on the levels of spontaneous MN in $Helq^{gt/gt}$ MEFs. The average percentages of binucleated cells positive for MN are shown. Experiments were repeated using different MEF lines so that 600 cells were observed per experimental group. (D) Shown are representative images of binucleated cells with MN (stained with DAPI, blue) that are either positive for CENP-A staining (CENP-A+, red) or negative for CENP-A staining (CENP-A-). White arrows point to MN. Scale bar is 10 µm. (E) Compared to WT cells, $Helq^{gt/gt}$, $Fancc^{-/-}$ and $Helq^{gt/gt};Fancc^{-/-}$ cells display an increase in both types of MN in untreated conditions (left), but an increase of CENP-A- MN in $Helq^{gt/gt}$ cells is not statistically significant. MMC treatment (600 nM for 2 hrs followed by 22-hr recovery) leads primarily to a higher number of CENP-A- MN (right). (F) The average percentages of binucleated cells

positive for MN are shown after CPT treatment (250nM for 6 hrs followed by 18-hr recovery). The levels for the untreated condition are duplicated on the right in gray for better comparison. Numbers in the white box show the increase above untreated conditions. Experiments were repeated at least three times using different MEF lines so that >600 cells were observed per experimental group. Error bars in (C, E, F) show the binomial error for the combined data set. Significance (determined by χ^2-test) at p<0.05, p<0.01, and p<0.001 are indicated as *, ** and ***, respectively. WT, $H^{gt/gt}$, $Fac^{-/-}$ and $H^{gt/gt}$;$Fac^{-/-}$ refer to wildtype, $Helq^{gt/gt}$, $Fancc^{-/-}$, and $Helq^{gt/gt}$;$Fancc^{-/-}$ respectively.

Supplementary Figure S7. $Helq^{gt/gt}$ cells do not display any significant changes in recombinant frequencies at the *FYDR* locus compared to wildtype cells. (A) The *FYDR* transgenic locus contains two tandem repeats of incomplete eYFP expression cassettes (jagged lines indicate deleted sequence information). An HR event at this locus can restore eYFP expression following either of two different methods of resolution: gene conversion as the result of a non-crossover event (top) or unequal sister chromatid exchange as the result of a crossover (bottom). Using wildtype and $Helq^{gt/gt}$ MEFs that carry this locus in the hemizygous state allows for the detection of HR events only during and/or after this locus has replicated. (B) Shown are sample flow cytometry plots from wildtype *FYDR* non-carrier MEFs (left) and wildtype hemizygous *FYDR* carrier MEFs (right). eYFP signals were detected using the FL1-H and FL2-H channels and counted using the “R2” gate. (C) The distributions of the number of $eYFP^+$ recombinants (per 10^6 cells analyzed) from are shown. Two samples were excluded from the data as outliers (number of recombinants in the untreated condition >25).

Cross	Number of pairs	Number of litters	Average litter size
Helq$^{gt/+}$ female x *Helq*$^{gt/+}$ male	4	5	7.6±0.75*
Helq$^{gt/+}$ female x *Helq*$^{gt/gt}$ male	6	15	6.3±0.62*

*No significant difference was observed between the average litter sizes by t-test.

Supplementary Table S1. *Helq*$^{gt/gt}$ males are fertile, producing about the same average litter size as *Helq*$^{gt/+}$ males.

Genotype	$Fancc^{+/+}$ or $Fancc^{+/-}$	$Fancc^{-/-}$	Total number	Expected number
Helq$^{gt/+}$	40	10	50	52.5
Helq$^{gt/gt}$	48	7	55	52.5
Total number	88	17*	105	
Expected number	78.75	26.25*		

* The observed number of 17 was significantly different from the expected number of 26.25 (p<0.05 χ^2-test).

Supplementary Table S2. *Helq*$^{gt/gt}$ mice are born in the expected Mendelian ratio but *Fancc*$^{-/-}$ mice show sub-lethality.
